# Poroid hidradenoma of the scalp in a US Veteran’s Administration (VA) patient

**DOI:** 10.1080/23320885.2021.1956933

**Published:** 2021-07-30

**Authors:** M. Mukit, M. Mitchell, I. Ortanca, N. Krassilnik, X. Jing

**Affiliations:** a Veterans Administration Medical Center Memphis, Memphis, TN, USA; b University of Tennessee Health Science Center, Memphis, TN, USA

**Keywords:** Scalp, poroid hidradenoma, Veteran’s Administration

## Abstract

Poroid hidradenomas are a rare subtype of hidradenoma. A Veteran’s Administration patient presented with a mobile, cystic scalp lesion. Intraoperatively the mass spontaneously ruptured. We hope clinicians will consider this entity on their differential when treating patients presenting with similar scalp lesions and intraoperative findings.

## Introduction

Poroid hidradenomas (PH) are a rare type of adnexal tumor. It is said that Abenoza and Ackerman were the first to describe them in 1990 [1]. Microscopic and immunohistochemical studies were subsequently done to further characterize this entity [2]. Poroid hidradenomas are a subtype of hidradenoma. Poroma and hidradenoma groups are interrelated, arising from a sweat duct, and sometimes show overlapping histological features. Hidradenomas divide into apocrine (‘clear cell’) and eccrine (‘poroid’) types. Other kinds of poroid neoplasms include eccrine poromas (EP), dermal duct tumors (DDT), and hidrocanthoma simplex (HS) [3]. These lesions have been described previously but mostly on a pathological basis. One histopathological series found only four poroid hidradenomas out of 18,653 biopsy specimens [3]. The largest case series reported fifty-six poroid hidradenomas but again, these specimens were mainly distinguished on a histologic basis [4]. These tumors arise from eccrine cells and are contained entirely in the dermis [5]. The risk of malignancy and metastasis is low but surgical excision is considered curative to prevent lesion growth and recurrence. Here we present the case of a scalp lesion in a VA patient with a non-descript clinical appearance, atypical surgical findings, and ultimate diagnosis of poroid hidradenoma on pathology.

## Methods

A patient was seen and evaluated at the VA Memphis plastic surgery clinic. After signing informed consent, he underwent surgical excision of a scalp lesion. A VA photo consent and release of information were obtained at his post-operative visit. The Memphis VAMC IRB determined this as a case study. The pathology slides were processed and were embedded in paraffin wax, sectioned, stained with hematoxylin and eosin, and examined and photographed under microscope magnification.

## Case report

A fifty-eight-year-old male with a history of hypertension, anxiety, depression, and chronic low back pain presented with a one-year history of a right parietal scalp mass. He believed that the mass first appeared after he bumped his head, but he was not sure. The patient denied any drainage, pain, numbness, tingling, fever, or redness. Medications included acetaminophen, bacitracin, ranitidine, ketotifen fumarate, ammonium lactate, castellani colorless topical paint, urea 20% cream, celecoxib, and cyclobenzaprine. The patient denied any smoking, alcohol, or drug use. Family history was non-contributory, and he had no known drug allergies. On physical exam, he was found to have a mobile and cystic appearing right parietal scalp mass with a punctum. The differential diagnosis included an epidermal inclusion cyst, pilar cyst, or lipoma.

**Figure 1.**
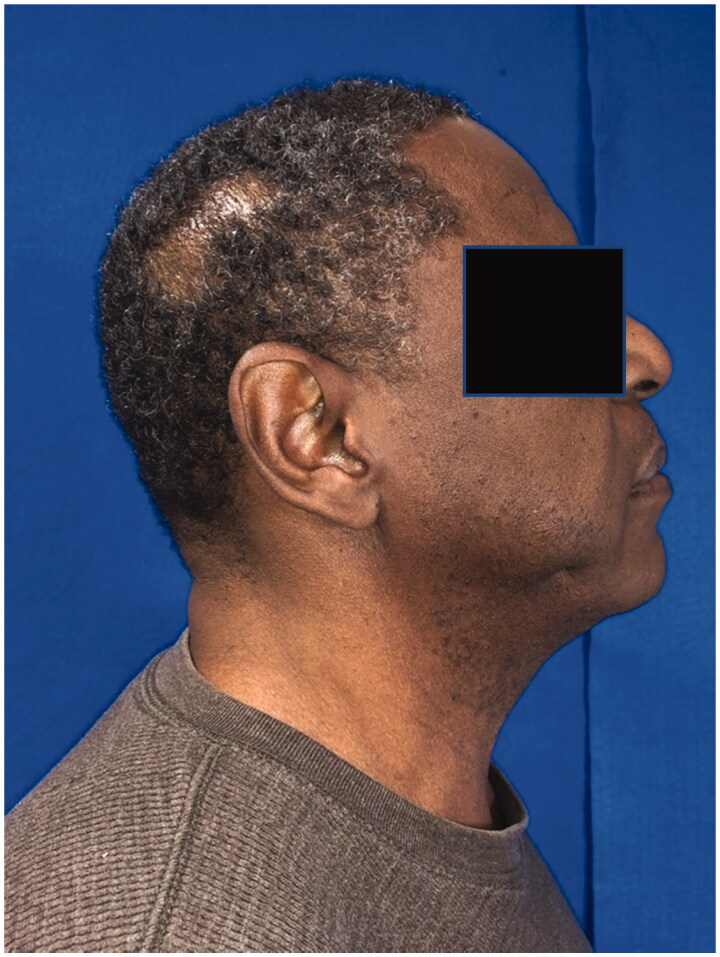
Lateral post-operative view of the right temporoparietal scalp.

**Figure 2.**
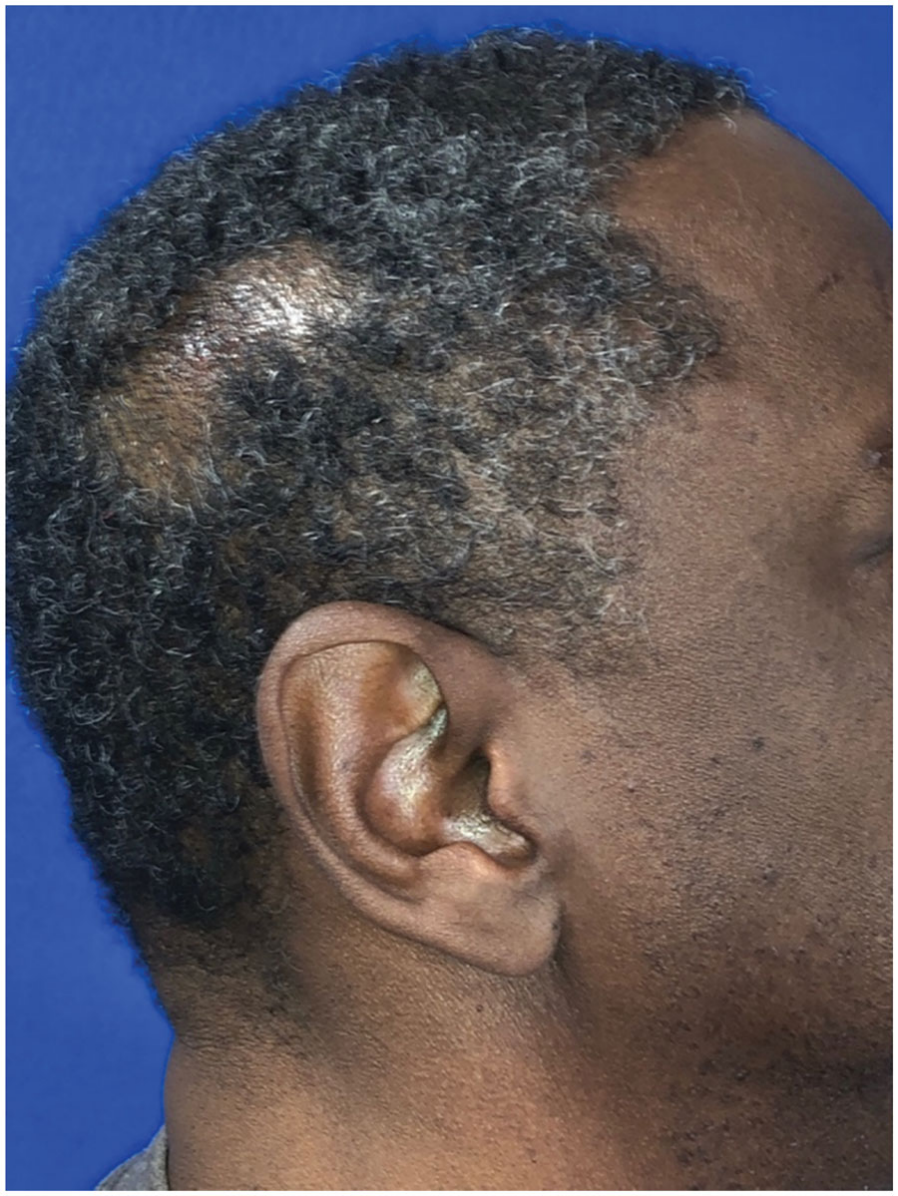
Close-up view of Figure 1.

**Figure 3.**
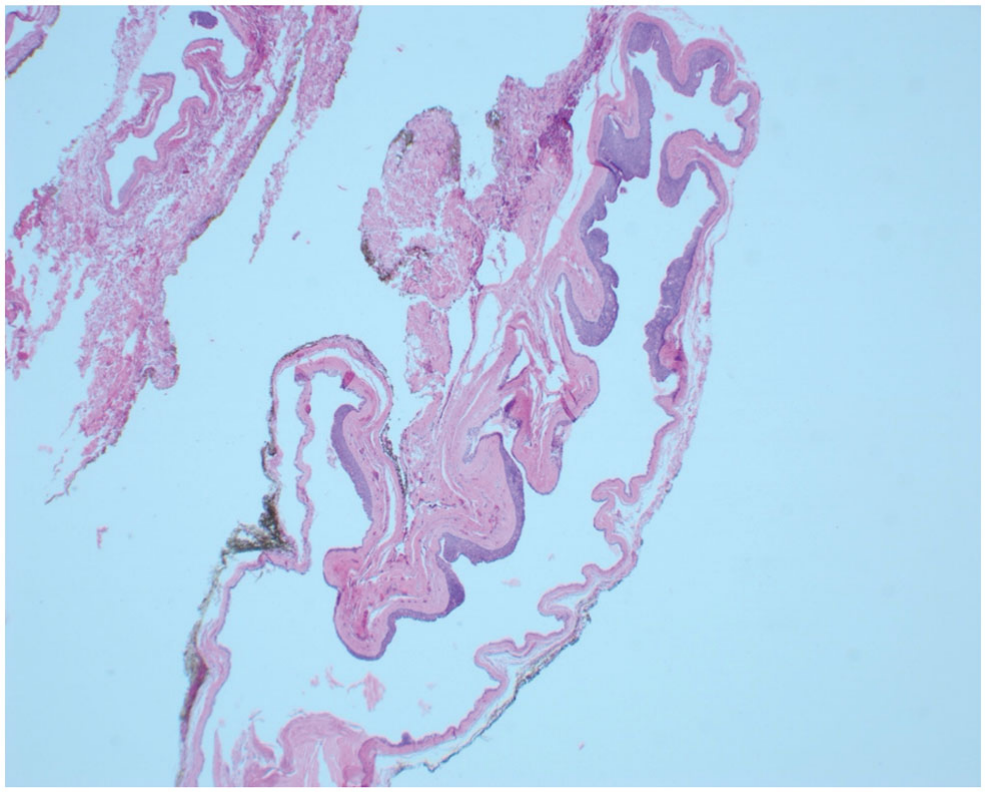
Low power micrograph of the lesion shows an extensive cystic component.

**Figure 4.**
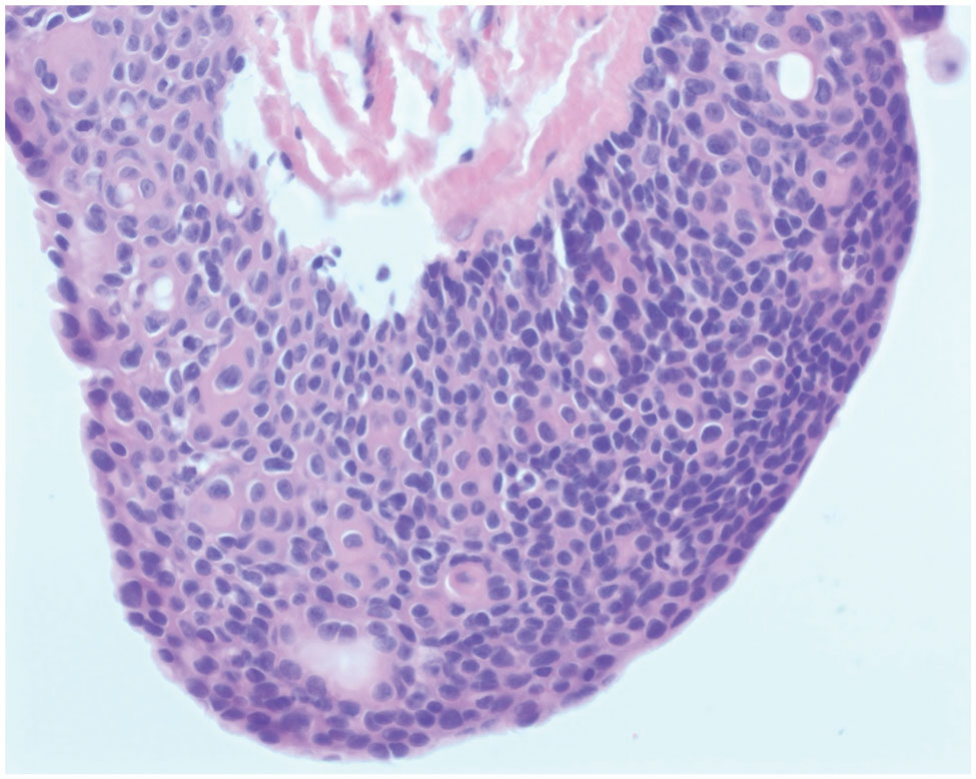
High power image of a solid area shows basaloid cells typical of poroma and occasional ductal structures (top right corner). The cells are uniform and cytologically bland. No mitotic figures are seen.

The patient underwent operative excision five days later under monitored anesthesia care. An elliptical incision was made around the lesion, including the punctum. The cyst was circumferentially dissected. It spontaneously ruptured and the specimen was passed off to pathology. The patient returned for a post-operative visit nine days later. He complained of right scalp incisional pain that was slowly improving but did not have any other issues. His staples were removed at that visit. Post-operative photos are displayed in Figures 1, 2. On pathology, the mass was measured to be 1.5 × 0.9 × 0.3 cm and described as pink/tan. The final diagnosis was ‘Fragments of an apocrine/poroid hidradenoma with a large cyst component. No overt malignant features seen’. Representative slides are shown in Figures 3, 4.

## Discussion

We add to the literature by describing a case of poroid hidradenoma in a United States veteran with a scalp lesion. Most clinicians do not consider this entity on their differential for scalp lesions. Poroid hidradenomas are an uncommon subtype of hidradenomas, which themselves are rare. Patients presenting with this condition range from ages 13 to 91 with the disease being more common in the sixth and seventh decades of life [6]. Some authors say that the incidence in males and females is about equal while others say that the male to female ratio is 3:1 [7,8].

Given the rarity of this entity, not much is known about the prognosis or treatment options. The prognosis is thought to be good as poroid hidradenomas are not known to progress to malignancy. Patients present for removal of this lesion secondary to pain, discomfort, aesthetic concerns, or concerns for malignancy [4–8]. A systematic review found that the preferred treatment is surgical excision; however, it is unknown whether other treatment options would work as they have not been explored [8]. Although one study suggested ultrasound may be useful in diagnosis—as these lesions have solid and cystic components *vs.* other entities like lipomas, which are homogenous—most lesions were excised without any pre-operative imaging or another workup other than clinical exam [3,4,6,7]. Due to the lack of information, there are no consensus guidelines or recommended surveillance regimens either. In their systematic review, Miller et al. recommended 6 months to 1 year follow-up since they did find one case of possible recurrence, and they recommended marginal excisions since there are no reports of malignant transformation [8].

On histology, poroid hidradenomas are lobular, circumscribed, non-encapsulated, solid, or partially cystic dermal tumors consisting of poroid and cuticular cells [3]. Poroid cells represent ‘miniature’ squamous cells, which are polygonal and have a higher nucleo-cytoplasmic ratio while cuticular cells form ductal structures within the tumor with eosinophilic cytoplastm [6].

Reports state that these lesions present as red or blue papules, nodules, or plaques, 1–2 cm in diameter, almost anywhere in the body—abdomen, trunk, neck, thigh, axilla, and limbs [6]. The tumor is reported to be well-circumscribed [7]. Poroid hidradenomas have been reported in the scalp, neck, arm, hand, thigh, back, and vulva [4,6–12]. This patient presented with the typical poroid cells usually found on histology for such lesions and his mass was within the typical size range.

Most of these lesions do not require reconstruction as they are typically 1–2 cm in diameter [7]. The scalp, with its laxity in the subgaleal plane, can be undermined to primarily close defects occurring secondary to tumor resection. Galeal scoring can be done as well, perpendicular to the line of tension, to increase flap length [13]. For our patient, we closed the dermis with 3-0 monocryl and the skin with skin staples. Larger scalp lesions may require local flaps. One case report detailed the use of a transposition flap with a skin graft to close the defect [14]. For larger defects, one could use local flaps, such as Oritrochea flaps, or regional flaps, such as the trapezius or latissimus dorsi to cover occipital wounds and temporoparietal fascial flaps to cover temporal wounds [13]. Alternatively, after temporizing a wound with a split-thickness skin graft, one could use tissue expansion to cover up to 50% of the scalp [13]. Defects that cannot be covered with local or regional laps may be reconstructed with free flaps, such as the latissimus dorsi flap, serratus anterior, omental flap, radial forearm fasciocutaneous flap, rectus abdominus flap, or anterolateral thigh flap [13].

Iblher et al. suggested a simple algorithm for reconstruction of scalp defects after oncologic defects that may be applied here: defects <3–4 cm may be closed primarily; defects <6–8 cm and <4–5 cm from the hair border may be reconstructed with some of the local flaps listed above [15]. Wounds <8–10 cm in size and the occipital region may be reconstructed with a regional flap and wounds in the temporal high frontal region may be reconstructed with a skin graft. Any larger wounds not meeting these criteria would need free flap reconstruction [15]. These lesions are not reported to get so large—in a systematic review, the largest one was 6 cm or so on an extremity and it was closed primarily—but larger lesions may be reconstructed *via* the mentioned methods [8].

Some studies suggest that these lesions are more common in immunosuppressed patients, such as liver transplant recipients and hematopoietic stem cell transplant recipients [4,16]. There are also case reports of lesions having features of both eccrine poromas and poroid hidradenomas [10,11].

With this case report, we hope to alert clinicians to the possibility of their patients having a poroid hidradenoma, especially if their patients have similar clinical histories and medical co-morbidities. As not much is known about these lesions, it is important that every poroid hidradenoma is noted and more data is obtained so that questions about prognosis, treatment, surveillance, and recurrence can be better addressed. Our case report is consistent with what others have found: poroid hidradenomas appear to be benign lesions with a good prognosis and are curable *via* surgical excision with low rates of long-term recurrence.
